# Measurement of lung clearance index (LCI2.5) by nitrogen multiple breath washout (N2-MBW) is feasible and well‍-‍tolerated by adults and children with cystic fibrosis

**DOI:** 10.1136/bmjresp-2025-003905

**Published:** 2026-04-03

**Authors:** Don S Urquhart, Emily J Taylor, Debbie Miller, Donna Bowens, Ellen Lacey, Mary Abkir, Clare J Saunders, Steve Cunningham, Zoe Louise Saynor, On behalf of the ExACT-CF study group, Don S Urquhart

**Affiliations:** 1 Department of Child Life and Health, The University of Edinburgh, Edinburgh, UK; 2 Department of Paediatric Respiratory and Sleep Medicine, Royal Hospital for Children and Young People, Edinburgh, UK; 3 Adult Cystic Fibrosis Unit, Western General Hospital, Edinburgh, UK; 4 University Hospitals Southampton NHS Foundation Trust, Southampton, UK; 5 National Heart and Lung Institute, Imperial College London, London, UK; 6 European Cystic Fibrosis Society, Lung Clearance Index Core Facility, London, UK; 7 Centre for Inflammation Research, The University of Edinburgh, Edinburgh, UK; 8 School of Health Sciences, Faculty of Environmental and Life Sciences, University of Southampton, Southampton, UK

**Keywords:** Cystic Fibrosis, Respiratory Measurement, Lung Physiology

## Abstract

**Background:**

Lung clearance index (LCI_2.5_), measured by nitrogen-multiple breath washout (N_2_-MBW), is a sensitive measure of ventilatory inhomogeneity that can be performed awake/unsedated from aged 3 years. However, concerns have been raised about the feasibility of LCI_2.5_ measurement by N_2_-MBW in adults with cystic fibrosis (CF), especially those with advanced lung disease, due to prolonged test durations. We assessed the feasibility, technical acceptability and patient perception of N_2_-MBW in adults and children/adolescents with CF within the exercise as an airway clearance technique-CF feasibility trial.

**Methods:**

N_2_-MBW (Exhalyzer-D, EcoMedics, Switzerland) was performed on two separate occasions, by multidisciplinary trial staff. Data were centrally over-read (European Cystic Fibrosis Society LCI Core Facility). Outcomes included the proportion of technically acceptable tests, numbers of trials per test, times per trial and total time to perform N_2_-MBW. Comparisons were made according to age group and forced expiratory volume in 1 s (FEV_1_) category (>70% vs <70% predicted). Participants also completed a questionnaire assessing acceptability and willingness to repeat testing.

**Results:**

49 participants (21 female; age: 10–55 years, FEV_1_: 38%–113% predicted) completed N_2_-MBW. Median (IQR) test durations were similar for adults (42 (27–55) min) and children (37 (26–44) min) with CF. Technically acceptable LCI_2.5_ results were obtained for 90/94 (95.8%) tests. Most participants rated the test easy to perform (87%) and comfortable (93%), with none reporting unwillingness to repeat N_2_-MBW testing.

**Conclusions:**

N_2_-MBW testing is feasible, technically acceptable and well-tolerated in people with CF across a wide age and disease severity spectrum. With appropriate standardised training and over-reading, LCI_2.5_ represents a robust outcome measure for consideration in CF clinical trials.

**Trial registration number:**

NCT05482048.

**WHAT IS ALREADY KNOWN ON THIS TOPIC**

- Lung clearance index (LCI_2.5_) measurement by nitrogen multiple breath washout (N_2_-MBW) is a sensitive measure of lung health.
- The utility of LCI_2.5_ in adults with cystic fibrosis has previously been questioned due to the time taken to undertake the test and its acceptability to adult patients.

**WHAT THIS STUDY ADDS**

- With standardised training and central over-reading support from respiratory physiologists, the measurement of LCI_2.5_ by N_2_-MBW can be successfully performed by multidisciplinary research staff (eg, nurses, physiotherapists) within a clinical trial setting.
- Test duration is comparable between adults and children, challenging assumptions that N_2_-MBW is impractical in older individuals or those with more advanced cystic fibrosis lung disease.

**HOW THIS STUDY MIGHT AFFECT RESEARCH, PRACTICE OR POLICY**

- Patient-reported experience of N_2_-MBW was overwhelmingly positive, supporting its use as a low-burden outcome measure in clinical trials.

## Introduction

 Lung clearance index (LCI_2.5_), measured by nitrogen-multiple breath washout (N_2_-MBW), is a non-invasive marker of ventilation inhomogeneity that is being increasingly used as a physiological endpoint in cystic fibrosis (CF) clinical trials.^1^,^4^ LCI_2.5_ quantifies the efficiency of gas mixing within the lungs by calculating the number of lung turnovers required to reduce the concentration of N_2_ to 1/40th (2.5%) of its baseline level. In simple terms, the LCI_2.5_ tells us how well air empties from the lungs and can highlight if some airways are emptying less well. N_2_-MBW testing requires the participant to initially breathe medical air (approximately 79% N_2_) to achieve a steady-state N_2_ concentration, followed by a switch to breathing 100% oxygen (0% N_2_), during which the N_2_ washout time and volumes are measured. LCI_2.5_ is determined across at least two N_2_-MBW trials.

LCI_2.5_ has a narrow normal range and can detect early lung disease, even when forced expiratory volume in 1 s (FEV_1_) is normal.^5^ As lung disease progresses, LCI_2.5_ increases, reflecting worsening ventilatory inhomogeneity. When compared with FEV_1_, LCI_2.5_ may be more sensitive to change following intervention,^6 7^ offering significant advantages for CF clinical trials, including smaller sample size requirements in this rare disease group. Moreover, its feasibility in young children further enhances its utility as an all-age trial endpoint.^6^

However, concerns remain regarding the utility of N_2_-MBW in adults, particularly those with advanced lung disease. Prolonged test durations have been reported in adult bronchiectasis cohorts,^8^ alongside low rates of technically acceptable trials ranging from 58% to 80% having been determined by central over-reading.^8 9^ Additionally, in those with severe lung disease, the tracer gas may not reach poorly ventilated areas of lung, leading to an underestimate of lung inhomogeneity. For adults with CF, N_2_-MBW has previously been described as time-consuming,^10^ uncomfortable and the least preferred test among a suite of monitoring tools (sputum sampling, spirometry, electrical impedance tomography and impedance oscillometry).^11^ Notably, much of this evidence predates the release of updated Spiroware software (V.3.3.1 and above), which has improved usability with the most commonly used Exhalyzer-D device.

A recent large study by Allomba and colleagues, using Spiroware V.3.3.1, suggests that N_2_-MBW is feasible in adults in a clinical setting.^12^ Building on this, we evaluated its application within a research setting through the exercise as an airway clearance technique (ExACT)-CF feasibility trial, which included children, adolescents and adults. Outside of industry-sponsored studies, there is limited evidence on LCI_2.5_ as a trial endpoint. In particular, data from investigator-led studies that employ centralised, standardised training alongside expert over-reading are sparse.^2^ The European CF Society Clinical Trials Network (ECFS-CTN) LCI Core Facility supports such standardised delivery for multicentre clinical trials (both CTIMPs and investigator-led).

This study aimed to evaluate the feasibility measured by technical success, test duration and patient acceptability of LCI_2.5_ measurement using N_2_-MBW in children, adolescents and adults with CF as part of the ExACT-CF feasibility trial,^13 14^ with ECFS-CTN LCI Core Facility support. We hypothesised that test burden and technical success may vary by age and site, and that adult participants may face greater challenges.

## Methods

This is a substudy assessing the technical acceptability, feasibility and acceptability of measuring LCI_2.5_ by N_2_-MBW in adults and children with CF under the care of teams in Edinburgh and Southampton (UK). The parent trial (ExACT-CF)^13 14^ was a randomised, pilot feasibility trial of ExACT where LCI_2.5_ was a key safety outcome.

### Training and measurement of LCI_2.5_ by N_2_-MBW

LCI_2.5_ was measured by N_2_-MBW (Exhalyzer-D device, EcoMedics AG, Duernten, Switzerland) at baseline and day 28. Trial staff delivering the tests included professionals from nursing, physiotherapy and physiology backgrounds. All underwent standardised training delivered by the ECFS-CTN LCI core facility at the Royal Brompton Hospital (London, UK). Data were analysed using Spiroware V.3.3.1 software^13^ and validated reference equations.^15^

### Technical acceptability of LCI_2.5_ measurement

Each test was centrally over-read for technical acceptability by the ECFS-CTN LCI core facility team (MA, CJS).

### Feasibility of LCI_2.5_ in adults and children

Feasibility was assessed through the analysis of a number of variables, averaged across baseline and follow-up testing. These included (1) the number of N_2_-MBW trials required, (2) the duration of pretest and active test phases and (3) the total time required to obtain three technically acceptable trials (of which two required successful over-reading).

Subgroup comparisons were then made between adults and children/adolescents, and differences by site and disease severity were also explored using non-parametric Mann-Whitney U tests (significance set at p<0.05).

### Acceptability of LCI_2.5_ to people with CF and clinical trial staff

Participants were invited to also complete a brief, bespoke questionnaire (see online supplemental material) assessing the ease, comfort, perceived time burden and willingness to repeat N_2_-MBW in the future; using a 5-point Likert scale. Free-text responses were also collated.

A purposive subsample of participants, caregivers and trial staff also undertook semistructured interviews exploring their trial experiences,^13 16^ including outcome measure perceptions. Interview transcripts were thematically analysed using a Framework Approach, and all content related to N_2_ -MBW extracted and summarised herein.

### Patient and public involvement

The ExACT-CF study was developed with extensive community involvement including several people with CF and their caregivers via the UK CF Trust patient involvement group, co-ordinated by Mrs Lorna Allen.

### Statistical analyses

Quantitative data were analysed using the IBM Statistical Package for the Social Sciences Statistics (V.25, IBM). Data are presented as median (range) or proportions, as appropriate. As most variables were not normally distributed, group comparisons were made using non-parametric Mann-Whitney U tests, with significance set at p<0.05. 95% CIs were calculated for between-group differences.

## Results

### Participant characteristics

50 participants were recruited to the ExACT-CF feasibility trial, of whom 48 participants (age: 10–55 years; FEV_1_: 43%–113% predicted) were randomised. Baseline demographics are summarised in table 1.

**Table 1:** Participant baseline demographics at randomisation (n=48)

Parameter	Distribution Mean (SD) or n (%)
Age (years)	20.5 (11.6)
Adults >16 years (n)	25
Children (<16 years (n)	23
Sex (n*,* male/female)	28/20
Height (m)	162.3 (14.3)
Weight (kg)	58.2 (18.2)
Body mass index (kg/m^2^)	21.7 (4.4)
Ethnicity (n*,* %)	
White–UK	44 (92%)
White–European	4 (8%)
CFTR genotype (n)	
F508del/F508del	28
F508del/other	20
Pancreatic insufficiency (n%)	42 (88%)
CF diabetes (n, %)	9 (19%)
LCI_2.5_	8.3 (2.9)
FEV_1_ (%predicted)	86 (19)
FVC (%predicted)	94 (13)

Values are means (SD) unless otherwise stated.

CF, cystic fibrosis; CFTR, CF transmembrane conductance regulator; FEV_1_, forced expiratory volume in 1 s; FVC, forced vital capacity; LCI_2.5_, lung clearance index.

### Technical acceptability of LCI_2.5_ measurement

LCI_2.5_ measurements were performed in 49 participants (one of whom exited the study prerandomisation) with paired baseline and day 28 data being available for 45 participants. Of 94 LCI_2.5_ tests undertaken, only 4 tests were deemed technically unacceptable by the ECFS-CTN LCI Core Facility. Comparisons between baseline and day 28 tests for the 43 participants with technically acceptable paired data are displayed in table 2.

**Table 2:** Feasibility and utility of LCI_2.5_ as a trial outcome (n=43 with baseline and day 28 N_2_-MBW data)

N_2_-MBW parameter	N_2_-MBW parameters stratified by lung function			N_2_-MBW parameters stratified by age		
	FEV1>70%	FEV_1_<70%	P value*	Adult (16+)	Child (<16 y)	P value*
Baseline LCI_2.5_	6.9 (5.9–11.8)	14.3 (6.3–16.9)	**0.002**	8.2 (6.0–16.9)	6.4 (5.9–14.3)	**0.001**
Number of LCI trials averaged over the two visits	5 (3–14)	4.5 (3-7)	0.69	4.5 (3–10)	5 (3–14)	0.16
Time for pre-measurement phase (seconds)	62 (37–128)	70 (51–90)	0.78	70 (43–106)	60 (37–128)	0.64
Time for active LCI measurement (seconds)	125 (75–258)	210 (50–388)	**0.02**	163 (92–388)	110 (50–188)	**<0.001**
Average number of breaths per trial (successful trials only)	40 (25–66)	71 (27–100)	0.14	46 (25–100)	39 (25–57)	**<0.05**
Total time to do N_2_-MBW (seconds)	2266 (1643–2742)	3371 (1018–5407)	0.21	2495 (1451–5407)	2280 (1018–3780)	0.22
Total time to do N_2_-MBW (minutes)	38 (27–46)	56 (20–90)		42 (24–90)	21 (17–63)	

* Median (range) displayed, Mann-Whitney U test.

FEV_1_, forced expiratory volume in 1 s; LCI, lung clearance index; N_2_-MBW, nitrogen-multiple breath washout.

### Feasibility of LCI_2.5_ in adults and children

49 individuals completed 94 tests of which 90 (96%) were technically acceptable. The median (IQR) age of those with acceptable tests was 16 (13, 25.5) years compared with 20.3 (14.5, 36.5) years in those with a test over-read as technically unacceptable (p=0.49). The median (IQR) FEV_1_ of those with acceptable tests was 89 (78, 101) %predicted compared with 92 (73, 109) %predicted in those with a test over-read as technically unacceptable (p=0.59).

**Figure 1:**
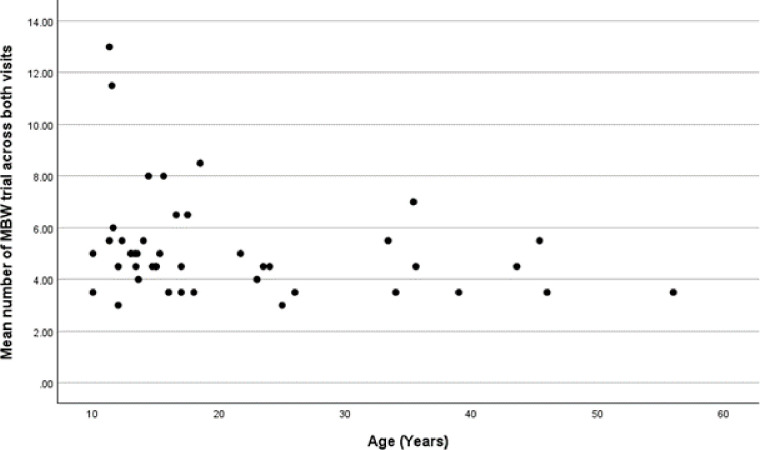
Number of N_2_-MBW trials required to achieve technically acceptable results by age. N_2_-MBW, nitrogen-multiple breath washout.

The time for each active washout trial was significantly longer in adults, at a median (IQR) of 163 (130, 214) seconds compared with 110 (95, 127) seconds in children (p<0.001). A median of 4.5 (3.5, 5.8) N_2_-MBW trials was undertaken in adults vs 5 (4.5, 5.5) trials in children (p=0.16). Figure 1 suggests that younger children may be required to undertake a greater number of N_2_-MBW trials in order to achieve technically acceptable results. As a result, total test time was not statistically different between adults who took a median (IQR) of 42 (27, 56) min and children who took 21 (27, 45) min in total to perform N_2_-MBW (p=0.22). These data are shown in table 2.

Those with worse CF lung disease (FEV _1_<70% predicted) had significantly longer average times per active washout trial at 210 (141, 383) seconds compared with 125 (103, 161) in those with FEV _1_>70% predicted (p=0.02). Those with FEV _1_<70% predicted took a median (IQR) of 56 (27-78) minutes to complete a N_2_-MBW test, while those with FEV _1_>70% predicted who took 38 (27–46) minutes in total to perform N_2_-MBW (p=0.21). These data are also displayed in table 2.

The small number of unacceptable tests (3/49 at Southampton, and 1/45 at Edinburgh) limited meaningful between-site comparison. However, the total time required to measure LCI_2.5_ did vary by site. In Edinburgh, a median (IQR) of 27 (25–35) min was taken to obtain technically acceptable LCI_2.5_ measurement, whereas in Southampton the test took 49 (39–60) min in total (p<0.001).

### Acceptability of LCI_2.5_ to adults and children with CF

Of the 48 participants randomised in the ExACT-CF feasibility trial, 15 (31%) returned the participant experience questionnaire (6 children/adolescents, 9 adults). Among respondents, 86.7% rated the test as ‘easy’ or ‘very easy’ to perform; 93.3% rated it ‘comfortable’ or ‘very comfortable’; and 100% reported willingness to repeat the test in the future.

It was acknowledged that if N_2_-MBW testing was new to the participant, the initial test time was longer due to trying to adapt to tidal breathing with a mask—“*I had never done the LCI before. So the first one was quite a lengthy one…the research nurse was like, oh, just breathe normally, so I was. And then she was like, oh, no, you need to breathe deeper, and suchlike, so the first one took a lot longer. When I went back the second time, it was done quicker, because I knew I needed to breathe a bit deeper than just breathing normally”* (adult with CF, aged in mid-40s years, male). For those familiar with the test, N_2_-MBW was felt to be a routine test*—“It was easy to do, it was like returning to somewhere that’s a familiar ground”* (parent of a young person with CF, aged in late 30s, female). The new software package was recognised as being much easier to use and cut down the length of test time, even for participants ‘with a lot of disease’ or moderate disease—“*There’s a new version of LCI (software) which is the first time I’ve used it, and that’s great. It just seems to cut down the length of time. So, even the ones who had quite a lot of disease, they still weren’t eight min long to try and wash out. They were much quicker, so I think that was good.”* (CF research nurse and N_2_-MBW operator, aged in late 50s, female). The support provided by the over-reading team was also commented on by trial staff. Furthermore, trial staff expressed a preference for more timely reading/feedback to highlight any errors or issues in order to avoid ongoing repetition of these errors*—“it’s just really nice to have that feedback within a week or so. And for your own improvement to make sure that that doesn’t happen again to highlight any issues that you don’t want to be continuously repeating”* (research nurse and N_2_-MBW operator aged early 40s, female).

## Discussion

This study evaluated the feasibility, technical performance and acceptability of LCI_2.5_ measurement by N_2_-MBW in a cohort of children, adolescents and adults with CF as part of a multicentre pilot feasibility clinical trial. With centralised training and oversight provided by the ECFS-CTN LCI Core Facility, a multidisciplinary group of trial staff, many of whom were new to N_2_-MBW, achieved a high proportion (96%) of technically acceptable LCI_2.5_ measurements across a cohort of participants with a wide range of age and disease severity.

As expected, participants with more advanced lung disease (typically adults) demonstrated higher LCI_2.5_ values than children. However, the time required to complete testing was similar between adults (longer washout as disease becomes more severe) and children (more trials, especially in younger children), despite prior reports suggesting N_2_-MBW may be prohibitively time-consuming in older or more severely affected populations.^8^,^10^

It was noted that there were between-site differences in test durations to achieve three acceptable trials. This is likely to be due to a combination of a) participant familiarity (the test is undertaken routinely in clinical practice for children in Edinburgh) as well as b) prior testing experience for N_2-_MBW operators—all Edinburgh tests were undertaken by an N_2-_MBW operator with more than 14 years’ experience.

Patient acceptability data were encouraging. Most participants rated the test as comfortable and easy to perform, with no individuals expressing unwillingness to repeat it in the future. These findings contrast with earlier reports describing N_2_-MBW as unfavourable in adults with CF,^10 11^ but concur with recent studies using Spiroware 3.3.1 software for N_2_-MBW where 92.5% success rates are reported for adults.^12^

We note that test durations in our study were similar to those previously deemed unacceptable by adults with bronchiectasis.^8 10^ It remains unclear why acceptability rates are higher in our cohort, but the adoption of the more usable (updated Spiroware V.3.3.1) software in our study, along with structured training for practitioners, may have mitigated some of the previous barriers to feasibility and patient satisfaction when using LCI_2.5_ as a clinical trial outcome. This is in line with recent observations from a large dataset in clinical practice.^12^

Limitations of this study include its modest sample size and the limited response rate to the participant experience questionnaire. We acknowledge that our small sample size and the use of univariate analyses may have introduced confounders; for example, comparisons between adults and children may be affected by factors other than age, such as lung function, infection status, etc. Although qualitative data enriched understanding of participant and staff perspectives, larger studies would enable subgroup analyses (eg, by age, disease severity) to broaden its generalisability. It is also noted that the study was conducted at two well-resourced CF centres within the UK CF Trust Clinical Trial Accelerator Platform network, both of whom have some existing N_2_-MBW experience, and that this may not reflect all CF sites. Replication in a broader range of trial settings is needed to confirm these findings and refine implementation strategies.

## Conclusions

Measurement of LCI_2.5_ by N_2_-MBW appears feasible across a wide range of ages (10–55 years) and levels of disease severity (FEV_1_ 43% to 113%predicted) in people with CF. Test durations were similar between children/adolescents and adults, and participant-reported experience was positive. These findings support the use of LCI_2.5_ as a candidate outcome measure for clinical trials involving both paediatric and adult populations with CF, while further work to establish the utility of LCI_2.5_ in routine clinical practice across the disease spectrum is also warranted.

## Supplementary material

10.1136/bmjresp-2025-003905 online supplemental file 1

## Data Availability

Data are available on reasonable request. All data relevant to the study are included in the article or uploaded as supplementary information.
