# Supplementary material for: Measurement of lung clearance index (LCI2.5) by nitrogen multiple breath washout (N2-MBW) is feasible and well‍-‍tolerated by adults and children with cystic fibrosis
Source: BMJ Open Respir Res. 2026 Apr 3;13(1):e003905. doi: 10.1136/bmjresp-2025-003905 (PMC13052781; doi:10.1136/bmjresp-2025-003905)
Supplement: online supplemental file 1 [file bmjresp-13-1-s001.docx]

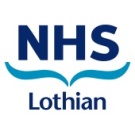
**SUPPLEMENTARY MATERIAL**

### LCI – Multiple Breath Washout Questionnaire

### We would be grateful if you would take a few minutes to complete this questionnaire in order to help us understand your experience with your lung function / breathing assessment using the LCI machine for Multiple Breath Washout.

### Please complete each section of this questionnaire by ticking the appropriate box.

**Age group of participant:**

10 – 15 16+

### LCI Test / Procedure:

|  | **Very Easy** | **Easy** | **Fair** | **Hard** | **Very Hard** |
| --- | --- | --- | --- | --- | --- |
| How did you find performing the test? |  |  |  |  |  |

|  | **Very Comfortable** | **Comfortable** | **Fair** | **Uncomfortable** | **Very Uncomfortable** |
| --- | --- | --- | --- | --- | --- |
| How comfortable did you find it to do the test? |  |  |  |  |  |

|  | **0-10 minutes** | **10-20 minutes** | **20-30 minutes** | **More than 30 minutes** |
| --- | --- | --- | --- | --- |
| How long did you feel the test took |  |  |  |  |

|  | **Yes** | **Maybe** | **No** |
| --- | --- | --- | --- |
| Would you be happy to do the test  again in the future? |  |  |  |

**Is there anything that could make things better for patients doing this test?**
